# “As long as the patient tells you it was a dog that bit him, why do you need to know more?” A qualitative study of how healthcare workers apply clinical guidelines to treat dog bite injuries in selected hospitals in Uganda

**DOI:** 10.1371/journal.pone.0254650

**Published:** 2021-07-14

**Authors:** Stevens Kisaka, Fredrick E. Makumbi, Samuel Majalija, Alexander Kagaha, S. M. Thumbi

**Affiliations:** 1 University of Nairobi Institute of Tropical and Infectious Diseases, Nairobi, Kenya; 2 School of Public Health, Makerere University, Kampala, Uganda; 3 College of Veterinary Medicine, Animal Resources and Biosecurity, Makerere University, Kampala, Uganda; 4 School of Public Health, University of the Witwatersrand, Johannesburg, South Africa; 5 Rabies Free Africa, Washington State University, Pullman, Washington, United States of America; 6 Paul G Allen School for Global Animal Health, Washington State University, Pullman, Washington, United States of America; Waikato Institute of Technology, NEW ZEALAND

## Abstract

Dog-mediated rabies is on the increase in Uganda despite the availability of post-exposure prophylaxis (PEP). PEP procedures are expounded in the Uganda Clinical Guidelines (UCG) of 2016. We assessed adherence by health workers to UCG while managing dog bites in two PEP centers and obtained insights into motivations of their practices. Using qualitative methods, we observed the health worker-patient encounters, reviewed medical records, and interviewed 14 health workers that were involved in managing dog bite injuries. We used deductive thematic analysis to identify codes in themes developed from UCG. We found that much of the history of the bites was taken, but it was neither verified nor written down on the patient’s file. Classification of wounds was inaccurate and ancillary laboratory assessments like culture and sensitivity tests were not conducted in all cases. Although antibiotics were given for both treatment and prophylactic purposes, the prescription was based on availability and affordability, not UCG recommendations. Rabies immunoglobulin (RIG) was not administered to deserving patients due to unavailability and high costs to the patient. Anti-rabies vaccine (ARV) was prescribed indiscriminately and some health workers attributed this to pressure from patients. Health education regarding prevention of dog bites was not given to patients due to time constraints on the side of the providers as a result of high caseloads at the emergency departments. Challenges to adherence to guidelines were identified as frequent ARV stock outs; inadequate cooperation among health facilities; and insufficient knowledge and skills on how injuries and rabies should be managed. We conclude that clinical management of dog bites is not fully in line with UCG. We argue that adoption of an integrated bite case management and cost-saving strategies as well as continuing medical education programs on rabies control and management could improve the clinical management of dog bites.

## Introduction

Rabies remains a global health challenge yet neglected in many societies. Globally, it is responsible for approximately 59,000 human deaths, 3.7 million disability-adjusted life years and USD 8.6 billion of economic losses annually [1]. The poorest regions of the world have the highest risk of rabies, with Africa and Asia annually contributing around 95% of the global cases. In such high-risk regions, there is low vaccination coverage of dogs, poor management systems for dogs, and ineffective policies on rabies [2]. It is estimated that 21,476 human deaths occur each year in Africa as a result of dog-mediated rabies [3].

Sustained mass vaccination programs of reservoir populations like dogs, and prompt administration of post-exposure prophylaxis (PEP) to dog bite victims are effective in managing rabies and preventing deaths [1, 4]. These two methods have been used to effectively control rabies in developed countries [5]. However, in Africa, there are challenges to implementation of such programs for example, the region spends the least on PEP [2]. Nonetheless, it is feasible that with better access to PEP and reduced prevalence of dog-mediated rabies, such deaths could be stopped. In fact, worldwide, PEP saves an estimated three million people that would otherwise have died from rabies annually [1]. This is because appropriate and timely PEP is almost 100% effective in preventing progression to rabies [6]. Given this opportunity, the World Health Organization (WHO) generally recommends immediate and thorough wound washing, vaccination with rabies vaccine, and in some cases, administration of rabies immunoglobulin (RIG) for dog bite victims [7, 8]. Uganda, a country with over 30,000 animal bites and an estimated per capita annual death rate (from rabies) of 0.39/100,000 [1], adopted the WHO recommendations in the Uganda Clinical Guidelines (UCG) of 2016 [8].

The UCG specify what healthcare workers should do for cases of rabies exposure. The key guidelines on clinical management of dog bite injuries in Uganda are summarized in S1 Table. Briefly, a series of five anti-rabies vaccine (ARV) doses on days 0, 3, 7, 14 and 28 is recommended for rabies PEP in those who are not previously vaccinated against rabies. Tetanus vaccination are recommended for those at risk of tetanus. Additionally, antibiotics are recommended for infected or high-risk wounds including: moderate to severe wounds; those presented with delays >8 hours; puncture wounds; wounds on hands, feet, or face; wounds with underlying structures involved; and wounds in immunocompromised patients. Importantly, the choice of antibiotics must be based on culture & sensitivity test results. Further, in case of clinical rabies, treatment must be started as soon as possible after exposure and should never be withheld from any exposed person whatever time interval has lapsed since exposure. However, despite the availability of such clinical guidelines, information on how they are being implemented for better patient care remains scarce. In this study, we assessed adherence to UCG on clinical management of dog bites in two PEP centers in Uganda. The aim was to examine the clinical management of dog bites and uncover ways of strengthening service delivery so as to improve patient care and treatment outcomes among dog bite victims.

## Methods

### Study sites

The study was conducted between March 2019 and October 2019 in two public healthcare facilities in Wakiso district and Kampala Capital City Authority (KCCA), in Uganda. These referral facilities were purposively selected because they provide PEP to most of the dog bite victims in the study area. One of the healthcare facilities serves Kampala City, which has an estimated human population of 1.5 million residents. It is estimated that 8% of households in Kampala own dogs at an average of nearly 2 dogs per household. The other healthcare facility serves Wakiso district which has a population of approximately 2 million people with around 14% of the households also owning dogs at an average of around 2 dogs per household [9]. Much as the healthcare facilities are located in their respective districts, dog bite patients from either district can report to any of the healthcare facilities to receive PEP. Each of the two healthcare facilities, located approximately 45 km apart, receives between 4–8 dog bite cases daily.

### Study participants

The primary participants of this study were health workers. In each healthcare facility, we identified several categories of healthcare workers who were directly involved in management of dog bite injuries. These included 7 clinicians (5 medical officers, 2 clinical officers) and 7 nurses. The clinicians were charged with the diagnosis of patients and prescription whereas the nurses were responsible for administering the prescribed treatment. Half of the participants had been involved in managing dog bites for at least 7 days prior to the interview.

### Data collection

#### Document reviews

We reviewed medical records of dog bite patients to understand their demographic characteristics of patients and the nature of the dog bite injury. These were also compared with the checklist developed from the UCG. The issues arising out of document reviews were later included in an in-depth interview questionnaire and followed up during the interviews.

#### In-depth interviews

In-depth interviews with the healthcare workers were conducted in English by the first author and two research assistants (RAs) using an interview guide developed by the authors (S1 File). The research assistants were carefully recruited based on demonstrated experience in qualitative data collection, and bachelor’s university level of education. The RAs were trained and oriented to the study purpose, rationale and data collection instruments, and ethical considerations. After the training, the research team pre-tested the in-depth interview guide on three healthcare workers attending to animal bite injuries in Mukono Health Center IV, which is outside the primary study area. Each of the interviews lasted for about an hour (50–70 minutes), was audio-recorded and then transcribed by an independent person. The first author reviewed each of the transcripts. Results from the pretests were used to refine the research instrument, share experiences from the field and draw lessons for the actual data collection exercise. During data collection, all the interview sessions were audio-recorded. In addition, summary field notes were taken during the interview to avoid loss of data in the event that the audio recorders failed to work.

### Data processes and analysis

**Fig 1:**
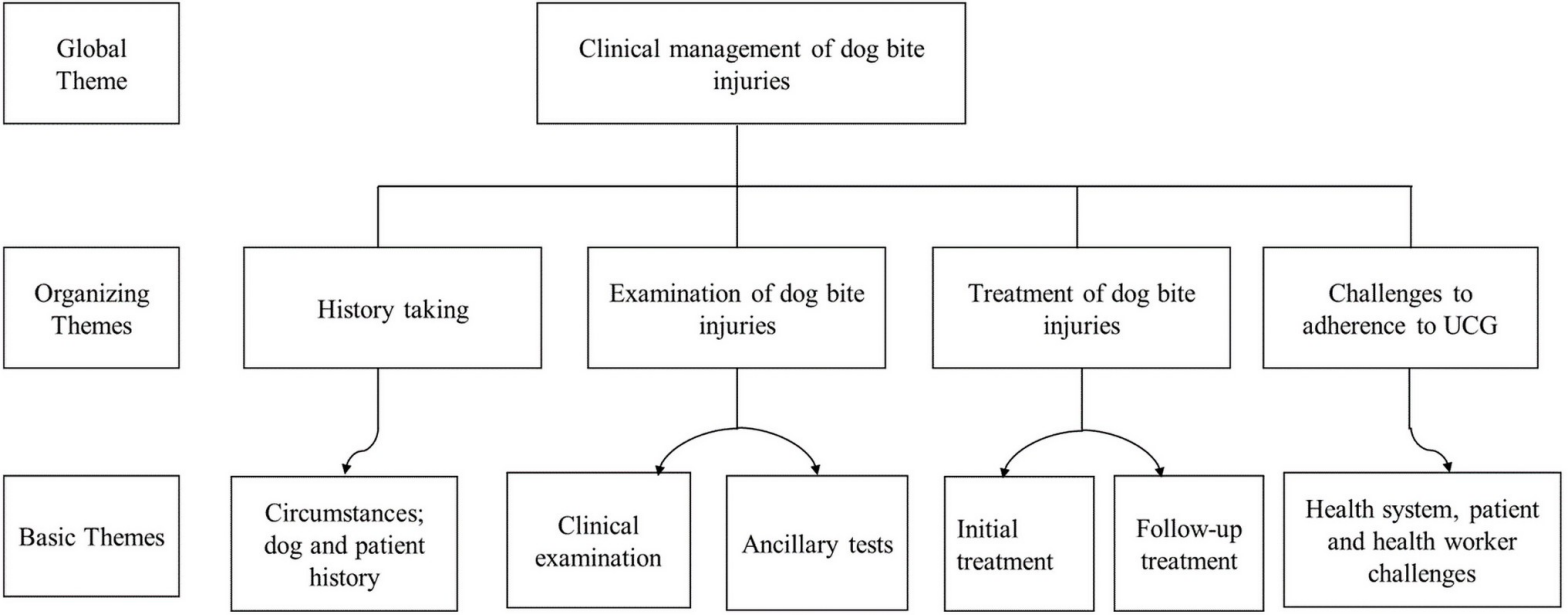
Thematic analysis of data on clinical management of dog bite injuries.

An independent team transcribed all audio recordings and the first author together with the two RAs thereafter proofread the transcripts. Errors of omissions and / or commission were detected and addressed during the reviews. Thereafter, unique identifiers were allocated to the healthcare facilities and transcripts with them aim of removing institutional and personal identifiers respectively. The transcripts were exported to N-Vivo 11 software and deductively coded following pre-conceived codes from the UCG. The broad theme was clinical management of dog bite injuries. This encompassed these organizing themes: History taking; Examination of the dog bite injuries; Treatment of dog bite injuries; Follow up of dog bite patients; and challenges to adherence to UCG as shown in Fig 1.

### Ethical considerations

Ethical approval was obtained for the study from the Mulago Hospital Research Ethics Committee (REF: MHREC 1518), and the Uganda National Council of Science and Technology (REF: SS4911). Written permission was obtained from the healthcare facilities prior to commencing the study. Before conducting observations and in-depth interviews, written informed consent was obtained from all the participants. Audio information was deleted after transcription and no personal identifiers were used in the report to ensure privacy and confidentiality of the information sources. Each of the respondents was given approximately USD 6 (20,000 Uganda shillings) as compensation for their time during interviews.

## Findings

The findings are presented according to the following themes and their corresponding concepts as shown in the in Table 1.

**Table 1:** Summary of themes and concepts that emerged out of the analysis.

Themes	Concepts
Taking the history of dog bite events	• History was being taken because it has links with the severity of the bite; treatment choices and outcomes.
	• History determines the risk of rabies of the biting dog in all cases.
	• There were variations in the depth of the history taken due to time constraints and high caseloads.
	• Rabies vaccination history was taken in very few cases because healthcare workers assume all victims are not vaccinated.
	• Previous dog bite episodes were not investigated.
	• History of tetanus vaccination was not investigated.
	• Much of the history is taken but it is neither verified nor written down on the
	• patient’s file.
Examination of the dog bite injuries	• Location of wounds was recorded in all cases although not all clinicians could accurately classify the bites.
	• Ancillary tests like radiology; complete blood counts; and culture and sensitivity test are not done.
Treatment of dog bite injuries	• Wound sanitation was undertaken in approximately one third of the cases.
	• Antibiotics are prescribed for treatment of infection and prophylactic purposes in three quarters of the cases but without sensitivity testing.
	• Tetanus vaccination was done in nearly three quarters of the cases.
	• Rabies immunoglobulin (RIG) is not given due to its unavailability and costs to the patient, even in the circumstances where it should have been given.
	• Anti-rabies vaccine (ARV) is at times given unnecessarily, for example in category I bites.
	• Health education regarding prevention of dog bites is not given to patients.
Follow up of dog bite patients	• Patients do not go back to the clinician but to the vaccination station where three elements are done: additional post exposure rabies vaccine doses; assessment of wounds; and reporting on the health status of the biting dog.
	• Non-compliance includes termination of treatment, violating the vaccination schedule and adding traditional treatments to the wounds.
Challenges in the clinical management of dog bites according to UCG	• Absence of the immunoglobulins; frequent stock outs of the vaccine; lack of collaboration and linkages among health facilities; distance to be covered by patients; high costs of treatment; deviations from wound home care instructions; and insufficient knowledge and skills on how rabies and dog bite injuries should be treated.

### Taking the history of dog bite events

#### Essence of taking history

Medical or case history of the patient refers to the information that a clinician obtains from a patient or their caretaker through asking questions, which are pertinent and specific to the history. History taking constitutes one of the key components of care for dog bite victims. Its major objective is to formulate a diagnosis to guide treatment [10]. This study explored the rationale for history taking through observations and in-depth interviews with clinicians. The findings reveal that in almost all the cases observed, the clinicians asked the patients about the history of the bite. They reasoned that knowing the history of the bite event has links with the severity of the event, treatment choices and likely outcomes:

*When patients come*, *there are those who will say I was bitten by a dog*. *Okay*! *Because when a person is bitten by a dog*, *you now get the history*, *when the dog bit the person*, *from where*, *then you ask is it a domestic dog*? *Is it a stray one*? *Do you know the owner of the dog*? *All those questions we ask because those questions help us in knowing how serious the case is and what type of drug to give and for how long*

*(R3*, *Medical Officer)*.

Therefore, history taking informs clinicians about the type, characteristics and health status of the dog. It is from this that the clinicians partly deduce the severity and seriousness of the bite and therefore choose the line treatment.

#### Determining the risk of rabies of biting dogs

In all cases observed, clinicians assessed the characteristics of biting dogs and circumstances of the bite as part of history taking. They particularly asked whether the dog was domestic or wandering; known to patients or not; and what the patient was doing before being bitten. Other circumstances investigated included whether the event was provoked or unprovoked. The respondents explained that such circumstances are essential in determining the dog’s rabid status as one explains:

*When they come*, *of course they are the ones to tell you “musawo* [healthcare worker] *I was bitten by a dog”*. *Then you ask them*: *Do you know the owner of the dog*? *Is it domestic or stray*? *Was it you who approached it or it came by itself to bite you*? *All this will inform me whether the dog is probably rabid or not*

*(R2*, *Medical Officer)*.

However, much as the UCG do not specify the parameters of history taking, it was observed that there were variations in the depth of the history taken. There were clinicians who were only interested in whether the patient was bitten by a wandering or domestic dog. They explained that the emergency departments have a high patient load and therefore, they have less time yet nearly all investigations result into administration of the ARV;

*As long as the patient tells you it was a dog that bit him*, *why do you need to know more*? *At the end of it all*, *whether they know the dog or not*, *you will have to give them the vaccine [anti-rabies]*. *So if the patients to see are many*, *that information is enough for me to decide*

*(R7*, *Clinical officer)*.

The interactions reveal that clinicians appreciated the need for investigating the circumstances of the bite. However, they identified time constraints and high patient loads in the emergency medicine department as the major reasons for obtaining less history. This may cause clinicians to indiscriminately prescribe ARV and failing to advise patients to observe the dogs since they cannot tell whether the biting dogs are available for quarantining or not.

#### Vaccination status of patient and prior treatments

The UCG advise that pre-exposure vaccination for rabies should be given to people at high risk of exposure to rabies, for example laboratory staff working with rabies virus, veterinarians, animal handlers, zoologists and residents in high risk areas. The guidelines also recommend different regimens for fully vaccinated and unvaccinated patients. However, it was noted that the clinicians inquired about the vaccination history of the patients in only 26/376 observations. On why the history of previous rabies vaccination was not regularly taken, clinicians explained that they assume all people are not vaccinated against rabies. In addition, some clinicians found it of no essence since even vaccinated patients for example veterinarians, insist on getting all the doses of the vaccine recommended for non-vaccinated people:

*Rabies vaccination is not routine*, *so almost all people are not vaccinated*. *Many times*, *we get the vets* [veterinarians] *bitten but they demand to get all doses*. *This includes those from wild life*. *So vaccination history is something I rarely ask*

*(R4*, *Medical Officer)*.

The presumption that every patient is not vaccinated at pre-exposure, coupled with the pressure to prescribe from patients infringes on the UCG. And because the UCG guides the treatment depending on whether one is previously vaccinated or not, not taking this history presents a likelihood of patients getting the ARV when they do not deserve them.

Further, the UCG implore clinicians to prescribe only two booster doses on days 0 and 3, for patients with full post-exposure rabies vaccination within the last 3 years [8]. This obviously requires the clinicians to investigate the history of prior bites and how they were managed, for each of the patients. However, in all 376 observations, none of the patients was asked whether they had had a dog bite episode before the current one. Nonetheless, upon being interviewed, only one practitioner agreed to having ever investigated this in practice. Others either found no need or it was something they did not think was relevant in practice.

*I rarely ask about that but I have received a few of such cases*. *Sometimes there are those who come and say that previously the dog bit them*, *they came here* [healthcare facility] *and completed the treatment*. *After 3 or 4 months*, *again the dog bites them and they come back*. *But according to the treatment* [guidelines], *if it is within one year*, *they are being protected*

*(R5*, *Medical Officer)*.

*No*. *I have not asked that question before*. *And even if they are there* [patients who had received post-exposure prophylaxis], *I do not think there is value in asking them because we are dealing with the current bite*. *So I take history on the current bite*

*(R2*, *Medical Officer)*.

From the above interactions, it can be seen that much as some clinicians know the importance of investigating this component, they just do not do it. This reveals a clear gap between knowledge and its application in practice. Secondly, it is evident that some clinicians do not realize the value in assessing this component of history. This points to the inadequate knowledge of UCG held by some clinicians as regards management of dog bites.

#### Time of bite

The time of the bite event is essential in determining whether the patient delayed to present for treatment or not. Delays have been previously associated with PEP failure as well as rabies-associated mortality [11, 12]. The UCG recommend that persons who present for PEP even months after having been bitten should be treated as if the exposure occurred recently. However, RIG can only be given up to 7 days post-bite [8]. In this study, it was observed that in all cases, clinicians asked about the time the event happened though this was to varying depths. In 141 out of 376 observations, there was effort to ask the patients about the estimated time of the bite. In the remaining cases, there were broad responses as regards the time, for example ‘yesterday’, ‘the day before yesterday’. The respondents reasoned that they investigate the time to evaluate the probability of success in preventing rabies, if the dog was indeed rabid.

*Finding out the delays is really important because of one aspect*. *For any clinical management*, *when you get early diagnosis and you get early treatment or intervention*, *the outcomes are better … For rabies per say*, *the incubation period is so varied that you find cases and you really wonder is it true or not*? *So*, *the time as to when they have reported and you have intervened by giving them the anti-rabies vaccine to develop their anti-bodies such that their bodies are immune is much more crucial for them*. *So*, *that is why history on time of bite is important*

*(R7*, *Clinical Officer)*.

Having investigated the time lag between the bite and seeking PEP, clinicians showed that they are aware of the essence of administering treatment within the required timelines.

#### Verification of history

Obtaining true history in PEP improves the accuracy of the diagnosis. However, in all observations made, the health workers did not make effort to verify what the patients were telling them. Even when some patients had carried vaccination cards of the dogs, the providers did not ask for them upon receiving the answer that the dog was vaccinated. Similarly, the prior rabies vaccination of patients was also not verified in all 26 cases that answered such a question in affirmative. Whereas some providers pegged their inability on huge caseloads (emergency medicine department handles other cases besides dog bites), others took it that patients know that accurate information is in their best interest and therefore no need to verify:

*Well*, *huh you can’t be sure of what they are telling you (laughs)*. *But you have to listen to the other party because you were not there*. *And you have to believe that the patient will be telling you the truth regarding his or her health*. *It is them that need the treatment*

*(R4*, *Medical Officer)*.

This shows that clinicians had relegated the responsibility of accurate history taking to the patients. Since history feeds directly into the diagnosis, there is a risk of unnecessary prescription of PEP or a decision not to prescribe PEP when it is indeed indicated.

#### Recording of history taken from dog bite patients

Much as a number of aspects of history were taken from the patient, there was little that was written down. A larger part of history was left as verbal in favor of what each clinician thought was is important:

*I do not write some things*. *I only write the necessary ones like the time when the dog bit the person*, *what sort of dog*, *is it a home dog*, *is it a stray dog which they have never seen*, *was it in someone’s compound tied or not tied*

*(R8*, *Clinical Officer)*.

However, because of this, nurses could not tailor the post-treatment counseling to a specific patient. Secondly, nurses could not explain to the patient why they were receiving a particular line of treatment:

*They bring the prescription to me*. *I give the treatment as requested*. *But I cannot specifically tell the patient what to do except the general things like wound care and compliance to treatment*. *Some are to get one dose of vaccine only*. *I can’t explain to the patient why but if the patients are not many*, *then I can ask the patient again if this was domestic dogs and I talk about observation of the dog for ten days*

*(R9*, *Nurse)*.

### Examination of dog bite wounds

During the examination of patients, it was observed that vital signs like body temperature, pulse rate, respiration rate and blood pressure were not taken. In addition, pain assessment was not done. However, in all observed cases, practitioners examined and assessed the bite wound.

#### Aspects of clinical examination of the site of bite

For all patients, there was a description of the location of the injury, for example face, hands, legs and others although no effort was made to take pictures for draw sketches for the patient’s file. All respondents explained the emphasis of examining the location:

*The site must be examined because there are some injuries*, *which are nearer the central nervous system*. *Those ones will not take long* [to develop clinical rabies] *because if the dog has rabies*, *it [virus] will move faster to the brain and they [patients] will get signs and symptoms of rabies*. *Therefore*, *the site of the bite is considered an emergency if it is near the central nervous system*

*(R5*, *Medical Officer)*.

#### Classification of the bite wound

For all observations made, there was effort to classify the bite wounds in either a standard way as per UCG or through a description. All respondents said that severity of the wound is important in choosing the treatment line. In what is conventionally category I wounds, there were two incidences where clinicians called their colleagues to determine if indeed there was no damage to the skin. Asked why, they explained that triangulation of examiners increases the accuracy in determining that the patient needs no medical treatment.

*Some people come with very intact skins and they claim I have been bitten*. *May be the dog did not manage to inflict a bite*. *I always get a colleague to help me by looking at the wound*. *If both of us cannot see something like an injury*, *then this is a category 1 bite*. *I just go ahead and counsel that patient*

*(R7*, *Clinical Officer)*.

However, all respondents could not accurately fit their descriptions of bite wounds into the right standard categories i.e. Category I (where dog licks on intact skin with no exposure); Category II (where there is nibbling of uncovered skin, minor scratches or abrasions without bleeding thus exposure); and Category III (with single or multiple transdermal bites or scratches and broken skin with saliva from dog licks thus severe exposure):

*Majority of the wounds are category I*. *By category I*, *I mean it is just a mere disturbance of the epidermis*, *they do not go deep into the flesh*. *Those that involve flesh and bone are rare*. *In most cases these constitute an emergency*, *severe bleeding*, *shock*, *a lot of anxiety*, *so those ones are managed on ward*

*(R3*, *Medical Officer)*.

On the importance of classification of severity of the wounds, all respondents agreed that it helps to guide the choice of treatment. In addition, some respondents said it guides in counseling the patients on what they should expect as regards wound healing as one explains below:

*Of course*, *you know how big the wound is*. *The bite can damage some of the internal structures around where the wound is*. *Like if it is calf muscles or joints*, *expect healing to delay*. *So you tell the patient that the wound is likely to stay for a longer time unlike in other bites*. *I also advise them to get some walking aid devices*

*(R2*, *Medical Officer)*.

#### Ancillary examination and testing

Clinicians mentioned that they go further to assess the wounds for infection by examining the injury for foreign materials, foul odor, erythema, exudates, edema and heat. They reasoned that this informs them on which antibiotic to use in either treating or prevention of infection.

*I check the site and how the wound looks like*, *how the patients are and the area*. *Because if the patient is coming from the village*, *there is always dust*. *Sometimes you suspect the wound may develop some infections or is already infected when it is hot*, *smalls bad or has fluids oozing out*. *This will tell you which antibiotic you should use*

*(R5*, *Medical officer)*.

*Sometimes you may suspect infection to have set in*. *When we suspect there is an infection*, *signs for infections are known like a fever and wound characteristics*. *So*, *when it comes to antibiotics*, *we give an antibiotic depending on the seriousness of the infection*

*(R7*, *Clinical officer)*.

Some clinicians had ever had an experience with rabid cases. In these circumstances, they said they undertake a differential diagnosis so that they rule out other causes of mental disturbance:

*You assess for any other cause*, *could it be cerebral malaria*, *it may be any other form of encephalitis*, *anxiety*. *So before I declare suspected rabies*, *I must have some level of authority so that I do not alarm the patient*. *Besides*, *I need to counsel the patient based on facts*

*(R2*, *Medical officer)*.

In all observations made, there was no recommendation for a wound culture or complete blood counts even when infection was suspected. The clinicians explained that because of the amount funds and time constraints involved in such tests, they usually forego them, even when they find some of them necessary:

*The vaccine in the causality [emergence medicine department] is free but gets finished up within a few days*. *So*, *you do not need to go into intensive investigations because patients can’t pay for them*. *But as a medic*, *you must know that history and visual inspection yields a lot on the dog bite*. *So you rely on that*

*(R2*, *Medical officer)*.

*Culture may be necessary but the time is limited*. *Patients come from far*, *so you cannot tell them to come back after a week to pick their results*. *It is better to give them an antibiotic that cover their infections broadly*

*(R5*, *Medical officer)*.

### Treatment interventions

In both study sites, the clinical management of dog bites comprises local wound sanitation, pain relief, antimicrobials, tetanus prophylaxis, and rabies prophylaxis. The clinicians who are diagnosing the patients are the ones that prescribe the line of treatment.

#### Wound management

In all observations, wound sanitation was prescribed in 132 cases as per UCG. These wounds were cleaned with either povidone iodine mixed with water; or soap and water. The clinicians explained that they recommend this in order to reduce the bacterial burden of the wound. They also advised the patients to continue doing the same at home after initial treatment, using locally prepared saline water. However, it was only very few cases that clinicians found the wounds worth debriding. Only one clinician had ever recommended debridement because the wound was too big in size; had the potential for infection; and was bleeding profusely. In this same case, the wound was closed mainly to control bleeding:

*In my practice*, *it was once*, *actually last year*. *We admitted one patient*, *they did debridement on him in theatre though*, *people were doubting whether it was a dog because the wound was really big*. *But he kept insisting that it was a dog*. *We had to clean*, *debride and actually closed the wound because it was big and it had bleeders*. *We actually closed it*. *That is the only one I have seen for debridement*

*(R4*, *Medical officer)*.

#### Use of antibiotics

In nearly three quarters of the observations, antibiotics were prescribed. Notably, in all cases that received antibiotics, there was no wound culture and sensitivity tests as per UCG. This was attributed to time constraints as well as the high costs to patients. However, the main reason for prescription of antibiotics was either to treat existing infections or to deter wounds from progressing to infection:

*Then there are infections like somebody who comes when they have placed other local medicine around it [wound]*, *definitely that you know that you have a potential infection and you have to prevent the infection that is likely to occur and then tell them to keep the wound clean afterwards*

*(R1*, *Medical officer)*.

*I check the site of the bite and how the wound looks like*. *But I also consider where the patient comes from and what they do*. *If it is a village*, *there is always dust*. *And you may find that this individual tells you they go to the garden often*. *If somebody has been working in the garden or goes there*, *you suspect the wound may develop some infections*. *You have to administer some antibiotics at least to prevent development of wound infections*

*(R7*, *Clinical officer)*.

#### Choice of antibiotics

For all observations, the antibiotics were limited to: amoxicillin, Ampiclox (ampicillin & cloxacillin), metronidazole, ceftriaxone, dicloxacillin, and Flucamox (amoxycillin & flucloxacillin). On what guides the choice of antibiotic, the clinicians cited “simplicity” of antibiotic, availability, affordability and progress of wound healing. However, even if treatment has been started, changes to the antibiotic could be made depending on wound healing and level of infection.

*Amoxicillin is a simple one I first prescribe*. *But there are times when the wound needs a stronger one for example when it is septic or turns septic*. *There I change to metronidazole for just 5 days*. *The patient will then continue cleaning the wound and eventually the wound will heal*

*(R6*, *Clinical officer)*.

*I choose the antibiotic depending on availability and someone’s pocket*, *but normally we try to give like the cloxacillin*, *amplicox*, *dicloxacillin*, *flucamox in that range and sometimes cefixime*. *I know the Ministry recommends some antibiotics but what is available is what you give even if it may be expensive for that [particular] patient*. *There*, *I write for them a cheaper one which they can get outside*

*[R5*, *Medical officer)*.

#### Tetanus vaccination

In the UCG, it is recommended that patients should receive a tetanus toxoid if the last tetanus vaccination was more than five years prior to the bite or for patients with unknown tetanus vaccination. Those with fewer than three lifetime vaccinations may receive tetanus immunoglobulin or tetanus toxoid. However, in all observations, the tetanus vaccination status was not investigated though the vaccine was prescribed in 298 cases. The clinicians explained that the decision to prescribe the tetanus vaccine depended on the low levels of hygiene of the wound, especially in those who attempt preclinical treatment:

*In most cases these dog bite wounds are not that bad*. *In a month you can get 1 or 2 with severe sepsis and there is severe contamination and you ask what have you been putting here*? *Someone obviously telling you cow dung or sisal bags*, *you anticipate that this dirty wound might have picked up a tetanus germ*. *But in most cases*, *people who come and tell you that when I got this problem* [dog bite wound], *I went to a local clinic or I washed here and it is a clean wound*. *Chances may be that you do not to give a tetanus toxoid in these circumstances*

*(R7*, *Clinical officer)*.

For those patients that did not received the tetanus vaccine, some clinicians attributed it to costs involved and rarity of the disease though all agreed that the vaccine is always available:

*The costs will be high when you add the tetanus vaccine*. *Patients won’t be able to afford……*. *But most of the times the tetanus vaccine is available*. *No mother at antenatal care goes without the tetanus vaccine*. *So it is available but I sometimes do not give it in dog bite patients due to costs*. *What is urgent and most important is the anti-rabies vaccine because tetanus is rare*

*(R2*, *Medical Officer)*.

#### Rabies immunoglobulin (RIG)

The UCG recommend RIG to be administered in cases where the biting dog’s vaccination records are not available, and can’t be restrained for observation (for example stray or wandering dogs) or is showing signs of rabies disease. In addition, RIG must be given in category III wounds or immunocompromised patients with category II wounds. However, for all observations, no RIG was prescribed. When asked why it was not prescribed at all, the clinicians said the health facilities do not stock it, including their private wings. They had resigned from ever prescribing it even for patients that could afford to purchase it:

*No*, *I have never given RIG because it is never available and it is also expensive for people to buy*. *If I can recall*, *I think immunoglobulin should be given when maybe somebody has been bitten by a dog and we suspect it to have the rabies*. *Honestly even if people can afford*, *I normally do not bother writing for them*. *It has never crossed my mind that an individual comes and has to get it*

*(R2*, *Medical officer)*.

*In xxxx* [name of facility], *we do not have human immunoglobulins for rabies*. *So*, *we do not have that and for years we have only managed with the rabies vaccine*. *The immunoglobulin part of it is expensive*, *we had people from India who wanted to give us* [supply] *the equine immunoglobulin but it was costly and remember most people who come are poor people*. *Do not forget that dog bites are a problem of the poor*. *So we rely on active immunization*. *That is what we do*

*(R6*, *Clinical officer)*.

However, some patients are said to demand for the RIG by themselves, though still it cannot be prescribed:

*Then some can come with preconceived information that we have the immunoglobulin and we should give [administer] it to them*. *These patients usually get information from internet*. *They come and say that RIG cures rabies*. *But still we counsel them out of it and give them a conviction that the vaccine will still do fine*

*(R7*, *Clinical officer)*.

One respondent described how a couple of years ago, the hospital got some few immunoglobulin doses for their private wing. However, the patients were not comfortable with the process of administering due to the pain at infiltration:

*There was a time we tried to bring it here but the patients did not receive it well*. *That was in the private wing*. *So*, *they were trying to add the immunoglobulin injecting it around the wound*. *The patient felt a lot pain and asked to be given only the vaccine*. *So*, *now we are only concentrating on the anti-rabies vaccine*

*(R1*, *Medical officer)*.

#### Management of pain

Analgesics were prescribed in 359/376 patients observed. They received either paracetamol ibuprofen or diclofenac. The clinicians explained that prescription of analgesics depends on whether the patient experiences pain or not:

*Not all patients get the pain killers*. *Some may come with a wound but they are not feeling any pain*, *so not everybody needs a pain reliever*

*(R3*, *Medical officer)*.

#### Anti-rabies vaccine (ARV)

Both study sites use the Abhayrab^®^ vaccine which is a purified vero cell rabies vaccine. It is a purified inactivated rabies vaccine prepared on Vero Cells using the L. Pasteur 2061/Vero Rabies Strain. The health facilities are using the updated Thai Red Cross (TRC) regimen where one dose of 0.1ml is given over each deltoid on days 0, 3, 7, 14 and 28. However, there are variations in the regimen depending on the type of the biting dog. Clinicians noted that the full set of 5 doses is given only in conditions where the dog cannot be observed for 10 days. For domestic dogs, including those with proof of rabies vaccination, the patients receive one dose at day 0 and then observe the dog:

*The other criteria where we give the first dose*, *we tell those people to observe the dog for 10 days*. *In the majority of cases*, *patients do not come back to give us report*, *meaning that the dog is fine and the patient is fine but we are not sure*

*(R6*, *Clinical officer)*.

*For domestic dogs which are vaccinated*, *I normally give one dose of PEP anti-rabies vaccine on day 0*. *On top of being sure that patient is guarded against rabies*, *the dose is also for psychological management pending observation*. *Therefore*, *this is done no matter the dog is vaccinated or not*. *We give it for a domestic dog*

*(R7*, *Clinical officer)*.

Nonetheless, the clinicians know that at times, the vaccine is given unnecessarily for example in category I bites. They attributed this to the high levels of anxiety that patients present with. In addition, they have reason to believe that the patients at times do not tell them right information in the hope that the practitioners will decide not to give the vaccine yet they want it:

*Then as regards to domestic dogs*, *some people who are too fearful*, *the hypochondriacs*, *they tend even to say no matter what the dog is vaccinated or not*, *vaccinate me if it has no harm*. *We can vaccinate such people*. *We can vaccinate them because they are willing and able to purchase the vaccine*. *You know our limitation to some vaccination is finance*

*(R2*, *Medical officer)*.

#### Management of rabies

One of the patients presented with clinical rabies during the study. The patient was referred to the national referral hospital where she was admitted. During admission of such patients, respondents said they stick to supportive treatment but the outcome is always negative:

*When one has clinical rabies*, *we can’t say “Mr*. *we are sorry you are going to die”*. *We admit them to lengthen their life pending death which is the only outcome in such cases*. *That’s why we admit them*

*(R7*, *Clinical officer)*.

However, there are some patients who know that the outcome will be bad and refuse to be admitted. In such cases, two of the clinicians said they allow them go home. Nonetheless, one of the clinicians said that before releasing them, the patient at least receives some symptomatic treatment, for example sedation, in order to restrain them on the journey home:

*Obviously*, *you can’t admit someone who doesn’t want*. *Me what I do is to counsel them*. *Then I sedate the patient with some diazepam to calm them down*. *I encourage them to manage the patient at their nearest health facility*. *So*, *that is what we do for those people who come when it is at a later stage*

*(R2*, *Medical officer)*.

#### Health education to dog bite patients

In both study sites, health education was being given after administration of PEP. However, this focused entirely on wound management including washing the wound; taking all the medication as prescribed for wound healing and pain management; dates of return; and what to expect while at home and during the return visit. This was done for all the 376 patients who were observed for this study. Notably, in both sites, there was no attempt to give the patients or their caretakers information on dog bite prevention. When asked why the health providers do not do this, they advanced reasons related to the high number of patients vis-à-vis the time required to attend to one patient as well as lack of health education materials:

*We have many patients to attend to*. *So I personally give the patients instructions on how to take their medicine*. *I have never given a talk on prevention of future bites to these patients or those who bring them*. *May be if there are flayers that we can give them*. *But in their absence*, *there is simply no time*

*(R9*, *Nursing officer)*.

### Follow up treatment and assessment

In both study sites, the three key elements that are of essence in follow-up visits are handled by the nurses. The three things include: additional post exposure rabies vaccine doses; assessment of wounds; and reporting on the health status of the biting dog. For patients who were to receive additional doses of the vaccine, they had to return on days 3, 7, 14 and 28. However, not all patients complied with the treatment calendar. For the observations made, all returned for the second and third dose whereas 371 and 242 came back for the 4^th^ and 5^th^ doses respectively. The nurses explained that some patients do not complete the doses because of treatment termination based on the nurses’ advice. This is in situations where the dog under observation is healthy after 10 days. Nonetheless, there are cases where the patients take it upon themselves to decide not to return for treatment because the dog is healthy as one nurse explains:

*Sometimes the patients feel they are okay and do not come back*. *This is usually when the dogs are okay and their wounds have healed*. *Those will not come back*, *not even to report about the dogs and finishing of antibiotics*

*(R14*, *Nursing officer)*.

#### Wound assessment on follow up

At the two study sites, the nurses examine the wound for progress to healing as well as infection. If there is infection, the nurse will refer the patient back to the clinician who prescribed the treatment for further advice. However, during the assessment process, they investigate what the patient has been doing for home wound care. If the home management is divergent from what was discussed on the prior visit, then more health education is done.

*There are those who come when they have applied Blackstone*, *beans*, *those drugs but we usually discourage them and say “do not do it again”*. *But they do it often and if infection has set in*, *we change and put them on stronger antibiotics*

*(R6*, *Clinical officer)*.

### Challenges related to healthcare workers’ adherence to clinical management of dog bites

This theme was organized under three challenges i.e. health system, patient and health provider challenges.

#### Health system-related challenges

As regards the health system, nearly all respondents concurred that absence of immunoglobulins was a major setback in managing human rabies cases. They reasoned that the presence of immunoglobulins in the UCG recommendations is testimony to their usefulness and therefore must be stocked.

*The Ministry* [of health] *argues us to use the immunoglobulins*. *But we have never received them*. *They only bring anti-rabies vaccines*. *If it is in the national guidelines*, *then it supposed to be supplied*. *Practice should not be different from the guidelines*

*(R2*, *Medical officer)*.

Another cross-cutting health system challenge from the health providers’ perspective were the vaccine stock-outs. While other medications are always in stock, the vaccine quantities, especially for the public wings of the health facilities, are inadequate. Those who couldn’t afford the vaccines in private wings missed their doses. It was observed that 251/376 (67%) accessed the vaccine privately while 57 patients went away without receiving the vaccine as prescribed.

*The problem is*, *others do not complete treatment because at times the vaccine is not available*. *So*, *if you tell them today there are no vaccines*, *some of them will be like we do not have money Musawo* [healthcare worker], *I will come back when it is available*. *Some keep checking for the free vaccine*, *others do not come back at all*

*(R11*, *Nursing officer)*.

Four respondents decried the lack of collaboration and linkages among health facilities. They explained that as the key treatment centers for dog bites, they ought to know each other’s capacity at a given time to aid referrals. For example, they said that before referring the patients due to stock outs of vaccines, they can’t establish the availability of the vaccine in the facility to which they are referring the patients. Secondly, for patients that come from far, respondents explained that it would improve compliance to treatment if follow up treatment is handled by the facilities where the patient comes from. However, there are no established ways of determining the different health facility capacities to handle dog bites.

*If a person is from Mbarara or Kabale* [places are over 250 km from this health facility], *there is no way I can crosscheck to find out whether the service is available so that I advise this patient to complete the treatment there*. *So*, *I have to tell the patient to come back here*. *But transport fares only are thrice the costs of treatment*

*(R9*, *Nursing officer)*.

*Because we do not have the contacts for our colleagues in xxxxx* [referral hospital], *we can’t ask them before referring our patients there*. *So*, *sometimes we send patients there and they return to say there is no vaccine too*. *The patient spends too much money looking for the vaccine*. *This is a serious problem to us too*. *Patients lose confidence in us*

*(R12*, *Nursing officer)*.

Respondents also identified distance as a key constraint to clinical management of dog bites. They said that distance not only translates into high treatment costs but some places are inaccessible and the programmed means of transport might not coincide with the scheduled days of the patient’s visit:

*Then some of them* [patients] *stay far like on the islands*. *Commuting from the islands and coming here is impossible on some days*. *And remember most dogs on islands are stray*, *not vaccinated*, *so they carry a high risk of rabies*. *But the challenge increases when they do not have the money to travel*. *If they come but have missed the schedule*, *you adjust the schedule dates accordingly and you encourage them to come*

*(R1*, *Medical officer)*.

#### Patient-related challenges

The patient-related challenges that the respondents discussed included non-adherence to the treatment schedule and recommended homecare management. All respondents said that they had ever experienced patients who had missed the scheduled doses. The reasons that the patients gave the healthcare providers included having travelled to far away areas for personal business; forgetting the schedule; lack of money for both transport and medical costs. For home care, some patients apply other substances to the wounds. Nearly all respondents had an experience of patients reporting having applied herbs and other substances to the wounds. They attribute this to patients seeking advice from other individuals who influence their choices. The respondents said they try as much as possible to counsel the patients out of such practices:

*Some patients apply traditional substances to the wounds even after receiving the initial doses of the vaccine*. *They report with beans tied on the wound*. *You ask them why and they say some relative advised like that*. *They mostly apply herbs and some Blackstone*. *Few come with them here but many just tell you they removed before coming here*

*(R14*, *Nursing officer)*.

#### Health provider-related challenges

The health provider challenge that came out of the discussion was the inadequate knowledge and skills in handling dog bites, especially among those administering the treatment. They explained that much as they are skilled in routine immunization (under the Uganda National Expanded Program on Immunization—UNEPI), dog bites are challenging because they are also handled in the immunization section. They explained that when they are new, they can’t give the required health education to the patients because they do not have experience on rabies vaccination.

*Knowledge is a big challenge especially when the rotation lands you here for the first time*. *That is a challenge*. *That is a challenge*. *That is a challenge*. *Unlike other immunizations*, *the rabies one is different*. *It needs you to know more about dog bites and dogs*. *It is not like the patient comes*, *you vaccinate and goes*. *Personally when I know there is a new person at the immunization wing*, *I go there regularly to teach them on how to handle animal bite patients*

*(R13*, *Nursing officer)*.

## Discussion

The key issues of the discussion are summarized in Table 2.

**Table 2:** Summary of key issues in the discussion.

Key findings	Discussion points and concerns
Insufficient bite and patient history	It has the potential to negatively affect risk assessment and may result into prescription of vaccines and antibiotics to those who actually do not deserve them.
Inconsistent risk assessment	This is a key cause of wastage of vaccines and incorrect patient care. Healthcare personnel should be trained further in risk assessment, if vaccines and medicines are to be used prudently.
Failure to undertake ancillary tests like culture and sensitivity.	Much as culture and sensitivity tests may be of no value for non-infected wounds, they are important in infected wounds. However, some researchers argue that if culture and sensitivity tests cannot be performed, empirical therapy based on amoxicillin and clavulanic acid may be used.
Indiscriminate prescription of rabies vaccines	It is mostly a result of inadequate risk assessment, pressure to prescribe from patients as well as lack of evidence-based practice. However, it is a key driver of high costs associated with PEP which typically constrain rabies control efforts in resource-limited settings.
Inadequate patient follow-up	There are no systems in place for the healthcare workers to contact patients to establish the results of observing the biting dog. Therefore, health workers do not even get to know the treatment outcomes when patients do not return to complete the treatment.
Unavailability of Rabies immunoglobulin (RIG)	This is a common constraint in rabies endemic settings which happen to be resource-limited. Given that RIG is increasingly becoming available and affordable, governments should facilitate healthcare facilities to stock some doses, at least for the severe-risk cases.
Inadequate health education and patient counseling	It deprives the dog bite patients of the opportunity for inspiration to complete treatment and promote better treatment outcomes. Health education and counseling should be promoted because they are important components of integrated bite case management.

This study assesses the practices of health workers with regard to clinical management of dog bite wounds. The study reveals the underlying reasons for the adherence as well as non-adherence to the recommendations in the Uganda Clinical Guidelines (UCG) that are pertinent to dog bite clinical management. It was observed that in both facilities, there was history taking; examination of the dog bite injuries; treatment of dog bite injuries; and follow up of dog bite patients. However, in each of the components, there were varying levels of deviations for example inadequate history, not recording or verifying the history; not classifying the bite wounds; prescribing antibiotics without sensitivity tests; and inadequate counseling of patients on homecare of the wounds. These gaps potentially compromise therapy as well as treatment outcomes.

The UCG recommend a through collection of epidemiological information on the history of exposure [8]. This is intended to aid rabies risk assessment for all patients who have been exposed to canine rabies. In addition, authors have argued that an appropriate clinical history guides the decision-making process for initial wound care, active or passive immunizations, obtaining cultures to determine specific pathogens as well as antibiotic prophylaxis and therapy [13]. In terms of history taking, it was observed that there was consistent effort to investigate how the bite occurred, duration since the event, whether the dog was provoked or not and vaccination status and whereabouts of the dog, if known. However, patient factors relating to risk of rabies, for example rabies vaccination, were not investigated in all patients. This opens up a gap for the clinicians to prescribe vaccines, which may actually not be required.

Not recording risk classification makes it difficult to establish the clinician’s basis for treatment. This is another potential source of indiscriminate PEP prescription whose consequences include vaccine shortages and reduced affordability. In Tanzania, it was found that if PEP were to be administered indiscriminately to animal bite patients, including to those with little or no possibility of genuine exposure to the virus, a threshold could be reached whereby PEP administration is no longer cost-effective [14]. We also noted that medical history taken misses assessment of co-morbidities (for example diabetes), medications (for example steroids), oncology interventions (for example radiation, chemotherapy), and life style habits (for example smoking and alcohol abuse) which are known to affect wound healing and infection risk [15, 16]. Alcoholism is particularly associated with increased susceptibility to infection by *Pasteurella* spps yet this bacteria is one of the commonest in dog bite injuries [17, 18]. Therefore, by not assessing them, clinicians cannot accurately identify patients who are suitable for prophylactic antibiotics. This may result into indiscriminate antibiotic use and its risks and increased costs of treatment to the patient.

Recommendations for post-exposure are dependent on the type of contact with the biting dog [19]. However, in all cases observed, the clinicians did not record the risk category. This may be explained by clinicians’ inability to rightly categorize the wounds because they could not correctly tell what category I and II entail. Some other studies have also found that low knowledge on risk classification; failure to record the risk category by clinicians after assessment of dog bite wounds; and general PEP, were rampant occurrences in Haiti [20], Turkey [21] and Bhutan [22]. Rabies risk assessment must be improved since Uganda is a rabies-endemic country where all dogs are taken to be potentially carrying the rabies virus.

Ancillary tests like culture and sensitivity test were not done in the study sites. When asked, the clinicians cited costs and time as barriers but there was no effort to investigate whether patients could afford or not. Failure to take tissue swabs or secretion samples for bacteriological culture is clearly against UCG which state that antibiotics should be prescribed after culture and sensitivity tests [8]. The likely consequence of this is the blanket prescription and administration of antibiotics even when it is not necessary. Much as initial cultures of non-infected dog bite wounds may have no value in predicting subsequent wound infection [23, 24], various authors have encouraged obtaining wound cultures to guide prescription for infected bites [17, 25].

The findings of this study on absence of culture and sensitivity tests is similar to another study on animal bite patients in Uganda where 77% were given antibiotics but all without sensitivity testing [26]. However, some authors argue that culturing the wound usually is only helpful if the wound has already abscessed or become infected [27]. They reason that swabbing a wound that is not infected results in the unnecessary identification and analysis of organisms which are colonizing the wound, rather than causing an infection. However, contrary to this, analysis of both clinically infected and non-infected dog to dog bite wounds have found them to be culture positive [28] much as pretreatment wound cultures are not predictive of bacterial species subsequently recovered from infected wounds [29]. Therefore, for non-infected wounds, the clinicians are probably right not to undertake sensitivity tests. For infected wounds, there are authors who propose that if culture and sensitivity are not available, empirical therapy based on amoxicillin and clavulanic acid may be used. This is because these antibiotics are active against most bite pathogens that can be isolated from bite wounds [30]. Nevertheless, this contravenes the UCG.

Dog bites have a potential to lead to tetanus infection. *Clostridium tetani* could come from the dog’s dirty oral cavity or the environment. In the study sites, tetanus vaccination status assessment was not routinely assessed in favor of rabies, although it is recommended in the UCG. Indeed, only one clinician assessed patients for the need for tetanus prophylaxis. However, in a country where the population serological immunity against tetanus is not known, the likelihood of tetanus should also be at the forefront. Besides, cases of tetanus following dog bites have been described in literature [31, 32]. In addition, situations where dog bite patients have presented with clinical tetanus rather than rabies have been documented [31]. This highlights the importance of the tetanus assessment in dog bites. Therefore, considerations for vaccines in cases of dog bites should routinely involve a thorough evaluation for the need for tetanus prophylaxis, especially those contaminated with soil.

For rabies prophylaxis, ARV was given to all patients assessed as having dog bite injuries, even if the risk wasn’t classified. This poses very few risks of rabies since all exposures are assured of getting the vaccine. However, this leads to unnecessary costs associated with PEP as observed in some studies [22, 33]. Further, just like some clinicians explained in this study, there is a tendency for patients to demand administration of the ARV. The fact that some clinicians comply with these demands means that there are prescriptions for ARV which are extracted under patient pressure. Studies have associated such patient demands with irrational prescribing and lack of evidence-based practice [34]. The fact that some clinicians capitulated to the pressure to prescribe resonated with findings of other studies [35]. Much as patient’s choice is important in the phenomenon of patient-centered-care, agreeing to inappropriate patient demands leads to wasteful costs in the face of limited resources for service delivery.

Prophylactic vaccination with ARV in the study sites follows the five-dose intramuscular (Essen) regimen administered on days 0, 3, 7, 14 and 28 into the deltoid muscle. This is inconformity with the revised guidelines which recommended the fifth dose to be given on day 28 hence dropping the dose on day 90 [36]. The adjustment of the regimen to terminate the vaccine after day 10 depending on the health of the biting dog is in line with the UCG [8]. A healthy dog after 10 days is reassurance that it is not infected with rabies [37]. However, the healthcare workers noted that some patients usually do not return to report the outcomes of the observation of the dog. Therefore, they cannot tell with confidence why such patients dropped out. Besides, there are no systems in place for the healthcare workers to contact patients to establish the results of the follow-up of the dog. This potentially presents a public health threat given that the health workers do not even get to know the treatment outcomes.

Rabies immunoglobulin (RIG) is not given due to its unavailability and costs to the patient, even in the circumstances where it should have been given. The UCG recommend giving RIG to all high risk rabies cases irrespective of the time between exposure and start of treatment [8]. However, even in cases that were bitten by dogs exhibiting pathognomonic rabies symptoms, the RIG wasn’t administered. This is similar to most rabies endemic countries where RIG is not regularly administered to deserving patients [38, 39]. Given that canine rabies is quite rare and RIG is increasingly available and affordable, healthcare facilities may be facilitated to stock a few doses for the severe-risk cases that need them most.

Health education is not done due to time constraints though they appreciated the importance of the educating the patient beyond just how to take the medication. Researchers have opined that animal bite patients should be inspired through health education at the time of initiation of vaccination in order to promote better treatment outcomes [40]. Since communities lack consistent rabies prevention and control programmes, this may be an opportunity to undertake some awareness. This may actually form a critical component of a rabies control program that may reduce the incidence of dog bites in high risk sub-populations. Studies have shown that that counseling animal patients about pet-related health hazards is important in reducing bites [41]. Indeed some countries have adopted a formal integrated bite case management (IBCM) programme to counsel animal-bite victims on the risk of rabies [42]. However, counseling patients may not necessarily improve treatment compliance as some authors have suggested [43].

The challenges to adherence as described by respondents were not unique; they cut across similar settings. Just like in this study, failure to administer immunoglobulins and ARV in India, Bangladesh Iran and Kenya has been described. In these countries, the failure has been attributed to stock outs as a result of lack of funds both at health system and patient levels [44–47]. An earlier study in Uganda also found out the same [26] meaning that years down the road, the situation has not changed much. Similarly, unsteady availability of PEP patients; inability to afford the cost of PEP; long distances to healthcare facilities; and poor healthcare seeking are frequent bottle necks in dog bite management in other developing countries [47–49]. Where the challenges are driven by lack of funds, cost-reducing strategies may be adopted in including adoption of the cheaper ARV dose-saving intradermal route. This may be complimented by incorporation of the rabies vaccination into routine vaccination programs, at least for high-risk sub-populations. Further, where there are problems of deviations from recommended wound home care, integrated bite case management (IBCM) may be adopted.

## Conclusions

The clinical management of dog bite injuries is not fully adherent to UCG. Generally, the health workers do not undertake the required steps in taking history and examining the injuries to assess the risk of rabies. There is also a high likelihood of unnecessary prescription of ARV and antibiotics for patients. The challenges experienced by clinicians during the treatment of dog bites are anchored in skills, time and funds. Therefore, we recommend adoption of integrated bite case management approaches and cost-saving strategies that can make clinical management of dog bites more efficient. For inadequate skills among healthcare providers, regular continuing medical education programs in rabies control and management is recommended.

## Supporting information

S1 Table Summary of guidelines on clinical management of dog bite injuries in Uganda. The table shows a summary of Uganda Clinical Guidelines on the clinical management of dog bite wounds. The key aspects of the guidelines include first aid, tetanus prophylaxis, prophylactic antibiotics, management of rabies and rabies post-exposure prophylaxis. The administration of anti-rabies vaccine and rabies immunoglobulins, together with how they are varied, is also shown in the table.
(PDF)

S1 File In-depth interview guide on adherence to clinical guidelines by medical workers during treatment of dog bite patients. (PDF)
