# Supplementary material for: “As long as the patient tells you it was a dog that bit him, why do you need to know more?” A qualitative study of how healthcare workers apply clinical guidelines to treat dog bite injuries in selected hospitals in Uganda
Source: PLoS One. 2021 Jul 14;16(7):e0254650. doi: 10.1371/journal.pone.0254650 (PMC8279313; doi:10.1371/journal.pone.0254650)
Supplement: S1 Table — The table shows a summary of Uganda Clinical Guidelines on the clinical management of dog bite wounds. The key aspects of the guidelines include first aid, tetanus prophylaxis, prophylactic antibiotics, management of rabies and rabies post-exposure prophylaxis. The administration of anti-rabies vaccine and rabies immunoglobulins, together with how they are varied, is also shown in the table. (PDF) [file pone.0254650.s001.pdf]

S1 Table: Summary of guidelines on clinical management of dog bite injuries in Uganda

The table shows a summary of Uganda Clinical Guidelines on the clinical management of dog bite wounds. The key aspects of the guidelines include first aid, tetanus prophylaxis, prophylactic antibiotics, management of rabies and rabies post-exposure prophylaxis. The administration of anti-rabies vaccine and rabies immunoglobulins, together with how they are varied, is also shown in the table.

First aid	Immediately clean the wound thoroughly with plenty of clean water and soap to remove any dirt or foreign bodies; Stop excessive bleeding; Rinse the wound and allow to dry; Apply an antiseptic*; Soak puncture wounds in antiseptic for 15 minutes; and Explore and debride (under local anesthesia, if possible).		
Tetanus prophylaxis	Give TT immunization (tetanus toxoid) if not previously immunized within the last 10 years.		
Prophylactic antibiotics	For deep puncture wounds; severe (deep, extensive) wounds; wounds on face, genitalia, and hands; and wounds in immunocompromised hosts. The choice of antibiotic must be based on culture and sensitivity test results. Do not use routine antibiotics for small uncomplicated dog bites/wounds.		
Management of rabies	Start as soon as the patient comes for medical attention, regardless of whatever time has passed from the exposure. Admit the case and provide palliative and supportive care. Observe hygienic precautions (use personal protective equipment) and counsel caregivers on rabies and consequences.		
Rabies post exposure prophylaxis	Local wound treatment: Prompt and thorough local treatment to reduce risk of infection.		
	Post-exposure vaccination in non-previously vaccinated patients: Give RV to all patients unvaccinated against rabies and in severe cases, give rabies immunoglobulin.		
	Post-exposure immunization in previously vaccinated patients: If vaccinated against rabies within the last 3 years, administer booster doses on days 0 and 3.		
	Passive immunization with rabies immunoglobulin (RIG): Give in all high risk rabies cases irrespective of the time between exposure and start of treatment but within 7 days of first vaccine.		
**RECOMMENDATIONS FOR RABIES VACCINATION/SERUM**			
**Nature of exposure**	**Condition of animal At time of exposure**	**10 days later**	**Recommended action**
Saliva in contact with skin but no skin lesion	Healthy	Healthy	Do not vaccinate
		Rabid	Vaccinate
	Suspect/Unknown	Healthy	Do not vaccinate
		Rabid	Vaccinate
		Unknown	Vaccinate
	Healthy	Healthy	Do not vaccinate

Saliva in contact with skin that has lesions, minor bites on trunk or proximal limbs		Rabid	Vaccinate
	Suspect/Unknown	Healthy	Vaccinate; but stop course if animal is healthy after 10 days
		Rabid	Vaccinate
		Unknown	Vaccinate
Saliva in contact with mucosae, serious bites (face, head, fingers or multiple bites)	Domestic or wild rabid animal or suspect		Vaccinate and give anti-rabies immunoglobulin
	Healthy domestic animal		Vaccinate but stop course if animal healthy after 10 days

*Chlorhexidine solution 0.05% or Povidone iodine solution 10%.
