# Supplementary material for: “As long as the patient tells you it was a dog that bit him, why do you need to know more?” A qualitative study of how healthcare workers apply clinical guidelines to treat dog bite injuries in selected hospitals in Uganda
Source: PLoS One. 2021 Jul 14;16(7):e0254650. doi: 10.1371/journal.pone.0254650 (PMC8279313; doi:10.1371/journal.pone.0254650)
Supplement: S1 File — (PDF) [file pone.0254650.s002.pdf]

S1 File. In-depth interview guide on adherence to clinical guidelines by medical workers during treatment of dog bite patients

"Good morning / good afternoon / good evening. I am **________** (introduce self).

This interview is being conducted to get to understand what you do when treating dog bite injuries in this health facility. I am especially interested in what motivates what you do when managing the dog bite wounds. If it is okay with you, I will be tape recording our conversation. The purpose of this is so that I can get all the details but at the same time be able to carry on an attentive conversation with you. I assure you that all your comments will remain confidential. I will be compiling a report which will contain all respondents' comments without any reference to individuals. If you agree to this interview and the tape recording, please sign the consent form as explained to you.

Ground rules

Before we start I would like to remind you that there are no right or wrong answers in this discussion. We are interested in knowing what you think, so please feel free to be frank and to share your point of view. It is very important that we hear your opinion.

1. What is your professional ground?
2. Are you vaccinated against rabies yourself? Describe.
3. For how long have you been treating dog bite patients? Take me through your experiences with dog bites patients. How do they present? What do you do for them?
4. What type of treatment do you give the patients?
5. Please describe in detail, the steps you undertake when a patient is presented to you.
6. What steps do you take to prevent infection of the dog bite wounds?
7. What type of therapy do you give patients to prevent progression to rabies? Describe.
8. What do Uganda Clinical Guidelines mention about management of dog bite wound? What circumstances prevent you from following the established guidelines?
9. Do you think it is important to inform patients if guidelines are not followed? Explain.
10. What may be the consequences of not following treatment guidelines?
11. Is there anything we have not discussed that you would want to talk about in line with management of dog bite injuries?

Thank you for taking the time to talk to me!!
